# Quadriceps tendon autograft for ACL reconstruction: A global survey from the International Quadriceps Tendon Interest Group

**DOI:** 10.1002/jeo2.70691

**Published:** 2026-03-25

**Authors:** Danko Dan Milinkovic, Riccardo D'Ambrosi, John Xerogeanes, Volker Musahl, Mirco Herbort, Christian Fink

**Affiliations:** ^1^ Center for Musculoskeletal Surgery Charité‐University Medicine Berlin Berlin Germany; ^2^ IRCCS Ospedale Galeazzi ‐ Sant'Ambrogio Milan Italy; ^3^ Dipartimento di Scienze Biomediche per la Salute University of Milan Milan Italy; ^4^ Department of Orthopaedics Emory University School of Medicine Atlanta Georgia USA; ^5^ Department of Orthopaedic Surgery UPMC Freddie Fu Sports Medicine Center, University of Pittsburgh Pittsburgh Pennsylvania USA; ^6^ OCM Clinic Munich Munich Germany; ^7^ Gelenkpunkt‐Sports and Joint Surgery, FIFA Medical Centre of Excellence Innsbruck Austria; ^8^ Research Unit for Orthopaedic Sports Medicine and Injury Prevention (OSMI), Private University for Health Sciences Medical Informatics and Technology Innsbruck Austria

**Keywords:** ACL, anterior cruciate ligament reconstruction, international survey, QT, quadriceps tendon autograft

## Abstract

**Purpose:**

To evaluate current preferences, techniques and indications regarding the use of the quadriceps tendon (QT) autograft for anterior cruciate ligament reconstruction (ACLR) among members of the International Quadriceps Tendon Interest (IQTI) group.

**Methods:**

An online survey consisting of 30 single‐ and multiple‐choice questions was distributed to all IQTI members (*n* = 45), representing an international group of orthopaedic surgeons with specific expertise in QT‐based ACLR. The survey was conducted in conjunction with the second IQTI meeting, and responses were collected over a 2‐month period (June–July 2025).

**Results:**

Thirty‐two high‐volume, experienced surgeons completed the questionnaire (32/45; response rate, 71%; 28 male and 4 female). The QT was the preferred primary graft choice for ACLR in 61% (*n* = 20) of respondents, while 89% (*n* = 28) used it for revision ACLR and 72% (*n* = 23) for multiligamentous injuries. Preoperative QT measurement with magnetic resonance imaging (MRI) or ultrasound was performed always or often by 34% (*n* = 11). Tendon defect closure after harvest was routinely performed by 94% (*n* = 30), and 33% (*n* = 11) reported always or often harvesting a bone block. Adjustable‐loop suspensory fixation was most commonly used on the femoral side (61%, *n* = 20), whereas tibial fixation was most frequently achieved with interference screws (72%, *n* = 23). One‐third of surgeons (33%, *n* = 11) reported modifying their rehabilitation protocol when using QT compared with other grafts. The most frequently observed short‐term complications were transient quadriceps weakness and extensor lag.

**Conclusion:**

This international survey shows that the QT autograft is a well‐established option in ACLR among experienced knee surgeons, particularly in revision and complex settings. At the same time, persistent variability in primary graft selection and reported rehabilitation and technical considerations indicate that QT use remains individualized within a spectrum of effective autograft choices.

**Level of Evidence:**

Level V, expert statement.

**Abbreviations**
ACL: anterior cruciate ligament
*AJSM*
: *American Journal of Sports Medicine*
ALCR: anterior cruciate ligament reconstruction
BPTB: bone–patellar tendon–bone
HT: hamstrings tendon
IQTI: International Quadriceps Tendon Interest Group
*KSSTA*
: *Knee Surgery, Sports Traumatology, Arthroscopy*
LEAP: lateral extra‐articular procedure(s)
MPFL: medial patellofemoral ligament
MRI: magnetic resonance imaging
PROMs: patient‐reported outcome measures
QT: quadriceps tendon
ROM: range of motion

## INTRODUCTION

The quadriceps tendon (QT) autograft has rapidly gained clinical acceptance as a primary option for anterior cruciate ligament reconstruction (ACLR) [3, 9, 12, 43, 51, 60, 61]. Once regarded as an alternative to hamstring tendon (HT) or bone–patellar tendon–bone (BPTB) grafts, the QT is now widely recognized for its favourable properties, intraoperative versatility and reliable clinical outcomes—leading to its expanded role in both primary and revision ACLR, particularly within individualized graft selection strategies [3, 41, 43, 54, 60, 61].

The QT offers several anatomical and functional advantages. Its robust cross‐sectional area, central alignment within the extensor mechanism and the option to harvest with or without a bone block contribute to its reproducibility and adaptability across diverse surgical scenarios [3, 4, 7, 9, 27, 39, 54, 59]. Biomechanical investigations have demonstrated comparable, and in some cases superior, properties relative to BPTB and HT grafts [1, 8, 24, 30, 32, 39, 43]. Clinical studies further support its efficacy in terms of graft survivorship, functional recovery and patient‐reported outcomes, with a potentially lower incidence of donor‐site morbidity and other complications compared with alternative grafts [28, 44, 48, 53, 55, 60, 61].

In 2018, the International Quadriceps Tendon Interest Group (IQTI) published a systematic review in the *British Journal of Sports Medicine*, advocating for broader adoption of the QT in ACLR [50]. That review summarized key anatomical, biomechanical and clinical considerations from the available literature and called for the orthopaedic community to systematically evaluate and implement QT‐based techniques. Since then, the global use of QT autografts has expanded substantially, accompanied by a growing body of literature and ongoing technical refinements.

To capture the evolving landscape of QT use, IQTI members conducted a structured expert survey during the second International IQTI Meeting held in June of 2025 in Munich, Germany. The meeting and subsequent survey aimed to consolidate current practice patterns, assess technical variability and highlight areas of consensus as well as ongoing controversy based on real‐world clinical experience. The primary purpose of this study was to systematically summarize current expert opinions on indications, surgical techniques, rehabilitation strategies and perceived limitations of QT graft use in ACLR.

We hypothesized that the QT is widely recognized by experienced knee surgeons as a valid and established graft option for ACLR, while acknowledging ongoing variability in technical details and areas of incomplete consensus.

## MATERIALS AND METHODS

The current study was determined to be exempt from institutional review board approval. Transparency In The reporting of Artificial Intelligence checklist (TITAN) checklist was fulfilled to transparently report the use of artificial intelligence.

A cross‐sectional survey was developed and distributed to all members of the IQTI and invited guests (*n* = 45), comprising orthopaedic and sports medicine surgeons with particularly high clinical and academic expertise in knee ligament reconstruction surgery.

The survey was administered through an online platform (Version 5.0; LimeSurvey GmbH) and remained open for responses between June and August 2025. It was initially designed by the IQTI ‘question group’ (M. H. and C. F.), reviewed by the ‘literature group’ based on a systematic review of the literature (R. A. and D. D. M.) and finalized through iterative discussion prior to the second IQTI Meeting. Participation in the study was voluntary and without remuneration. No email addresses or other identifying information were collected. Each participant could submit only one response per device.

The questionnaire consisted of 30 structured items, including single‐ and multiple‐choice formats, designed to comprehensively evaluate all important aspects of QT use in ACLR (Supporting Information S1: Appendix I). Questions were grouped into five domains: (1) Demographics (Q1–6), (2) Indications and Diagnostic Considerations (Q7–16), (3) Surgical Technique (Q17–24), (4) Rehabilitation and Postoperative Management (Q25–26) and (5) Complications and Limitations (Q27–30). Participants were asked to report their surgical preferences, frequency of QT graft use in primary versus revision ACLR, technical details of graft harvest and fixation and clinical scenarios in which QT was most commonly indicated. Additional items addressed imaging practices for preoperative planning, perceived complication profiles and rehabilitation protocols. Some questions were designed to allow multiple answers (Supporting Information S1: Appendix I).

### Statistical analysis

Survey results were analysed descriptively using frequency distributions and percentages. Absolute values (*n*) were calculated for each response based on the 32 participating surgeons. Data were processed in Microsoft Excel (Microsoft Corp., 2023) to generate descriptive statistics and graphical visualizations for each thematic section of the survey.

## RESULTS

A total of 32 out of 45 members of the IQTI group and invited guests who were asked to participate completed the survey, resulting in a 71% response rate.

### Demographics

**Figure 1:**
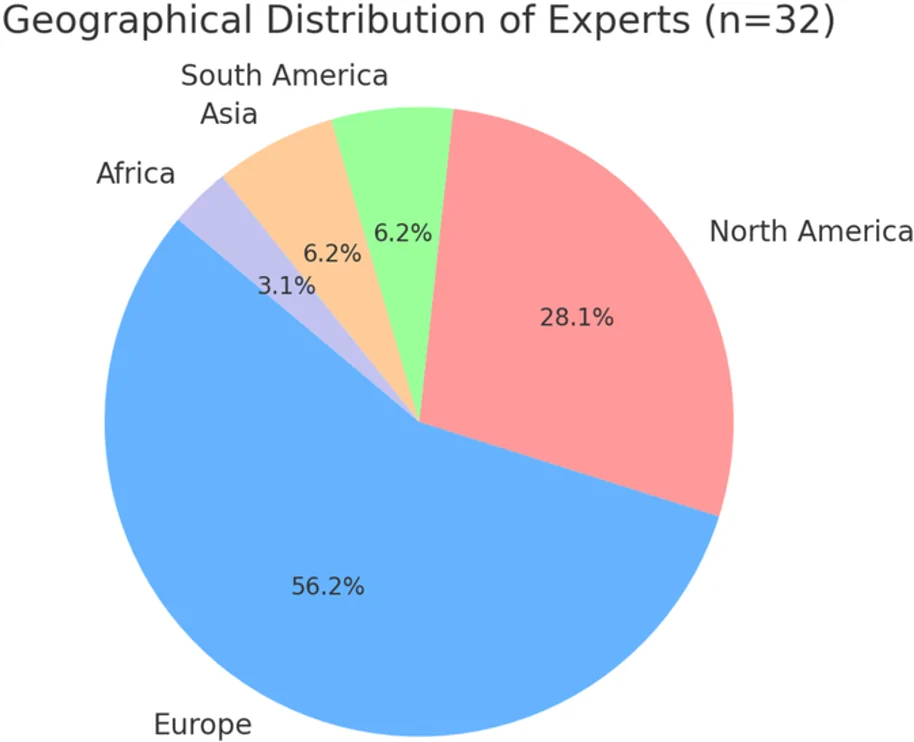
Geographical distribution of experts.

**Figure 2:**
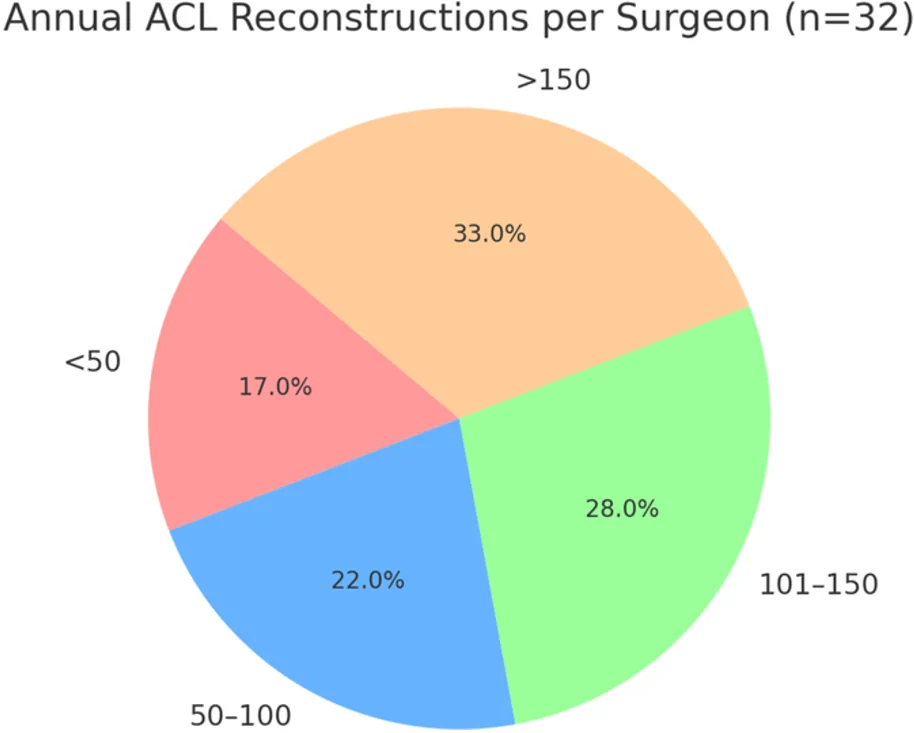
Annual ACL reconstructions per surgeon. ACL, anterior cruciate ligament.

Most respondents were between 45 and 55 years of age (*n* = 20, 61%), followed by 35–45 years (*n* = 7, 22%), >55 (*n* = 4, 11%) and 2 members <35 years of age (6%). Regarding surgical volume, 33% (*n* = 11) performed >150 ACLRs per year, 28% (*n* = 9) between 100 and 150, 22% (*n* = 7) between 50 and 100 and 17% (*n* = 5) <50 ACLRs per year. There were 18 experts from Europe (56.3%), 9 from North America (28.1%), 2 from South America (6.3%), 2 from Asia (6.3%) and 1 from Africa (3.1%) who completed the survey. The male‐to‐female ratio was 28:4 (Figures 1 and 2).

### Indication and diagnostic considerations

**Figure 3:**
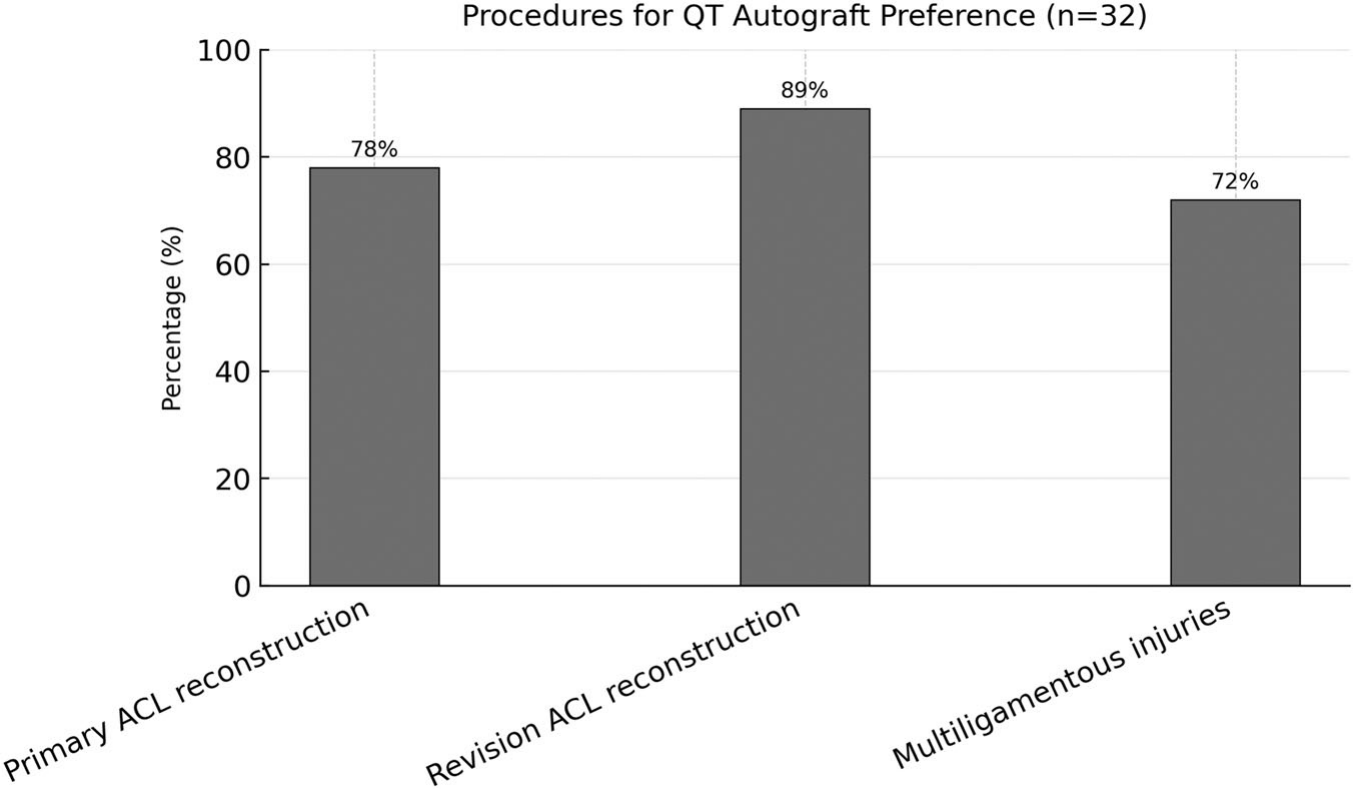
Preference for the use of QT autograft in various procedures. ACL, anterior cruciate ligament; QT, quadriceps tendon.

The QT was the preferred graft for ACLR among 61% (*n* = 20) of respondents, with none selecting patellar tendon or allograft. The majority reported using QT in revision ACLR (89%, *n* = 28), primary ACLR (78%, *n* = 25) and multiligamentous injuries (72%, *n* = 23) (Figure 3). In their own practice, 33% (*n* = 11) used QT in 76%–100% of primary ACLRs, 28% (*n* = 9) in 51%–75%, 22% (*n* = 7) in 26%–50% and 17% (*n* = 5) in <25%. Regarding patient selection, 39% (*n* = 12) used QT in all patients, 39% (*n* = 12) in active athletes, 39% (*n* = 12) in female football players and 17% (*n* = 5) in alpine skiers.

For skeletally immature patients, 28% (*n* = 9) reported always using QT, 17% (*n* = 5) often, 50% (*n* = 16) sometimes and 6% (*n* = 2) never. The most cited advantages of QT were biomechanical properties (100%, *n* = 32), morphology (78%, *n* = 25) and availability (61%, *n* = 20). Key demographic considerations included level of activity (89%, *n* = 28), age (78%, *n* = 25) and profession (78%, *n* = 25), while sex (44%, *n* = 14), BMI (22%, *n* = 7) and nicotine abuse (6%, *n* = 2) were less relevant.

Important clinical factors included extensor apparatus strength (44%, *n* = 14), tibial slope (39%, *n* = 12), dynamic valgus (39%, *n* = 12) and leg axis (33%, *n* = 11). Main contraindications were prior QT injury (94%, *n* = 30), quadriceps atrophy or neuromuscular dysfunction (89%, *n* = 28) and poor tissue quality or scarring (67%, *n* = 21).

### Surgical technique

Preoperative imaging of QT thickness via magnetic resonance imaging (MRI) or ultrasound was performed always by 28% (*n* = 9), often by 6% (*n* = 2), sometimes by 39% (*n* = 12) and never by 28% (*n* = 9). When imaging was done, cross‐sectional area was considered most important by 44% (*n* = 14), thickness 3 cm above the patella by 39% (*n* = 12) and length by 28% (*n* = 9).

Vertical skin incision was preferred by 67% (*n* = 21), horizontal by 33% (*n* = 11). The tendon was harvested using free‐hand technique in 56% (*n* = 18) and harvesting instruments in 44% (*n* = 14). Regarding the periosteal flap, 28% (*n* = 9) always harvested it, 28% (*n* = 9) often, 22% (*n* = 7) sometimes and 22% (*n* = 7) never. After tendon harvesting, 94% (*n* = 30) always closed the defect, while 6% (*n* = 2) never did. Harvest with a bone block was performed always by 11% (*n* = 4), often by 22% (*n* = 7), sometimes by 33% (*n* = 11) and never by 33% (*n* = 11).

**Figure 4:**
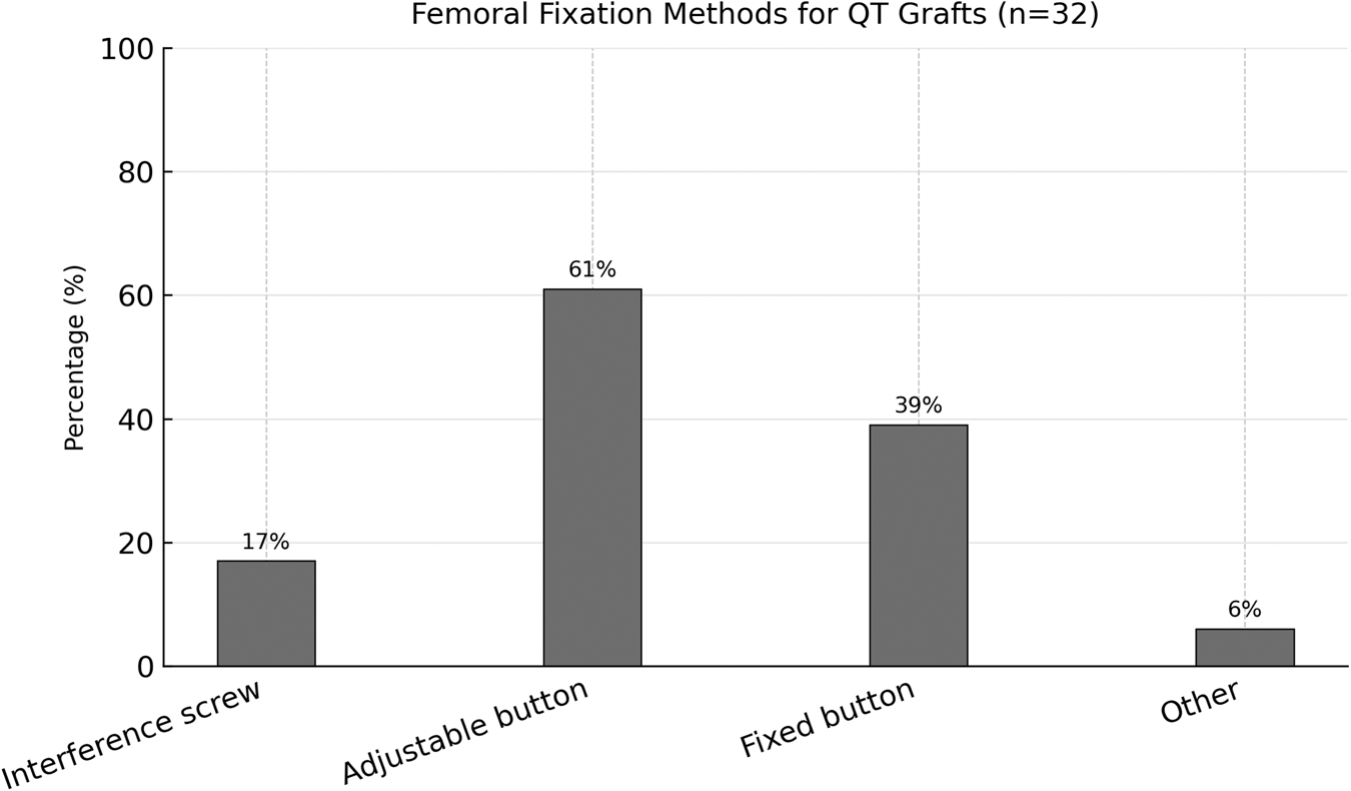
Preference for various femoral fixation methods. QT, quadriceps tendon.

The femoral fixation method was most frequently an adjustable button (61%, *n* = 20), followed by fixed button (17%, *n* = 5) and interference screw (17%, *n* = 5) and other (6%, *n* = 2) (Figure 4).

**Figure 5:**
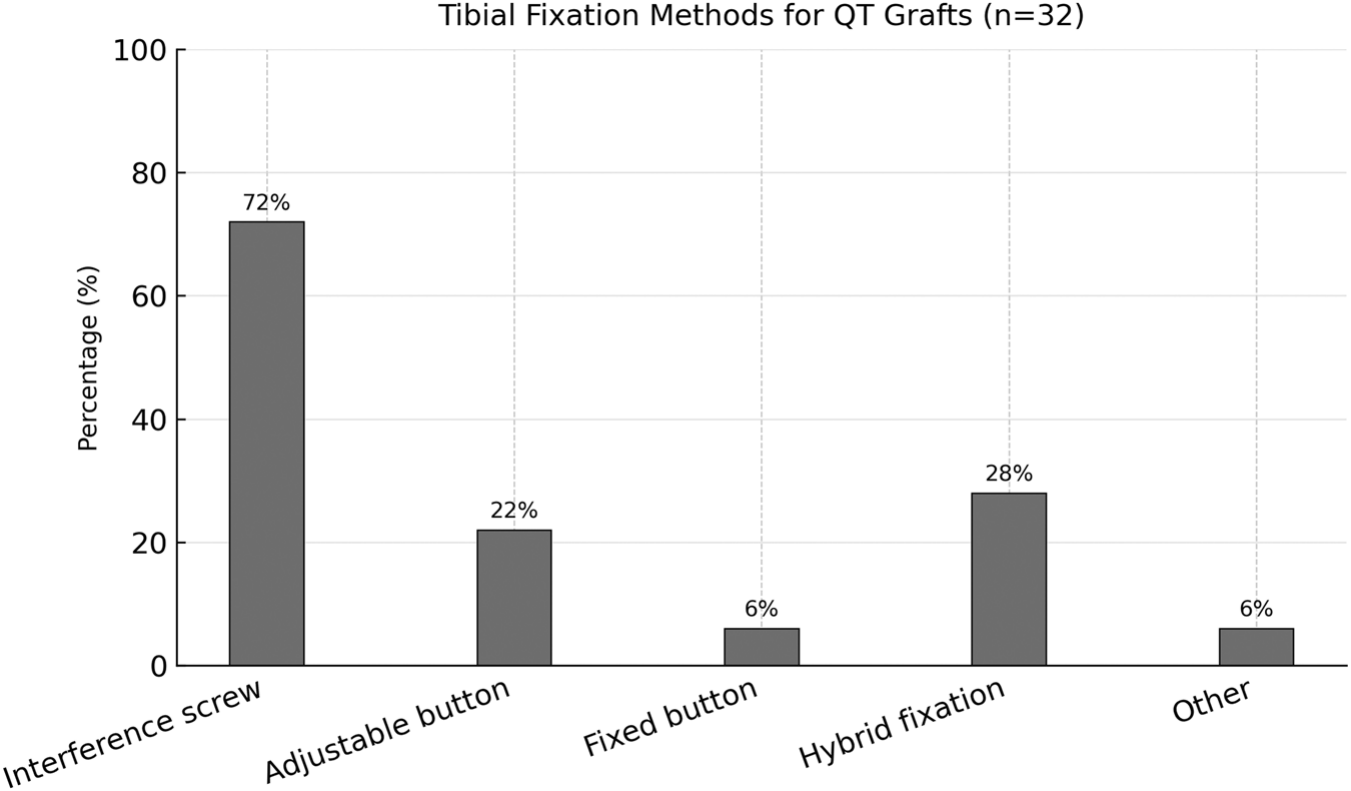
Preference for various tibial fixation methods. QT, quadriceps tendon.

On the tibial side, fixation was most commonly performed with interference screws (72%, *n* = 23), hybrid methods (28%, *n* = 9), adjustable button (22%, *n* = 7) or fixed button (6%, *n* = 2) (Figure 5). Here, we must emphasize that multiple answers were allowed for various options.

Regarding lateral extra‐articular procedures (LEAP), 33% (*n* = 11) performed them in 0%–25% of ACLRs, 50% (*n* = 16) in 26%–50%, 11% (*n* = 4) in 51%–75% and 6% (*n* = 2) in 76%–100%.

### Rehabilitation

One‐third (33%, *n* = 11) of participants modified their rehabilitation protocol when using QT grafts. Reported adaptations included modified range of motion (ROM) (33%, *n* = 11) and extended immobilization (11%, *n* = 4); 67% (*n* = 21) selected ‘Other’, while no respondent reported altered weight‐bearing.

### Complications and limitations

**Figure 6:**
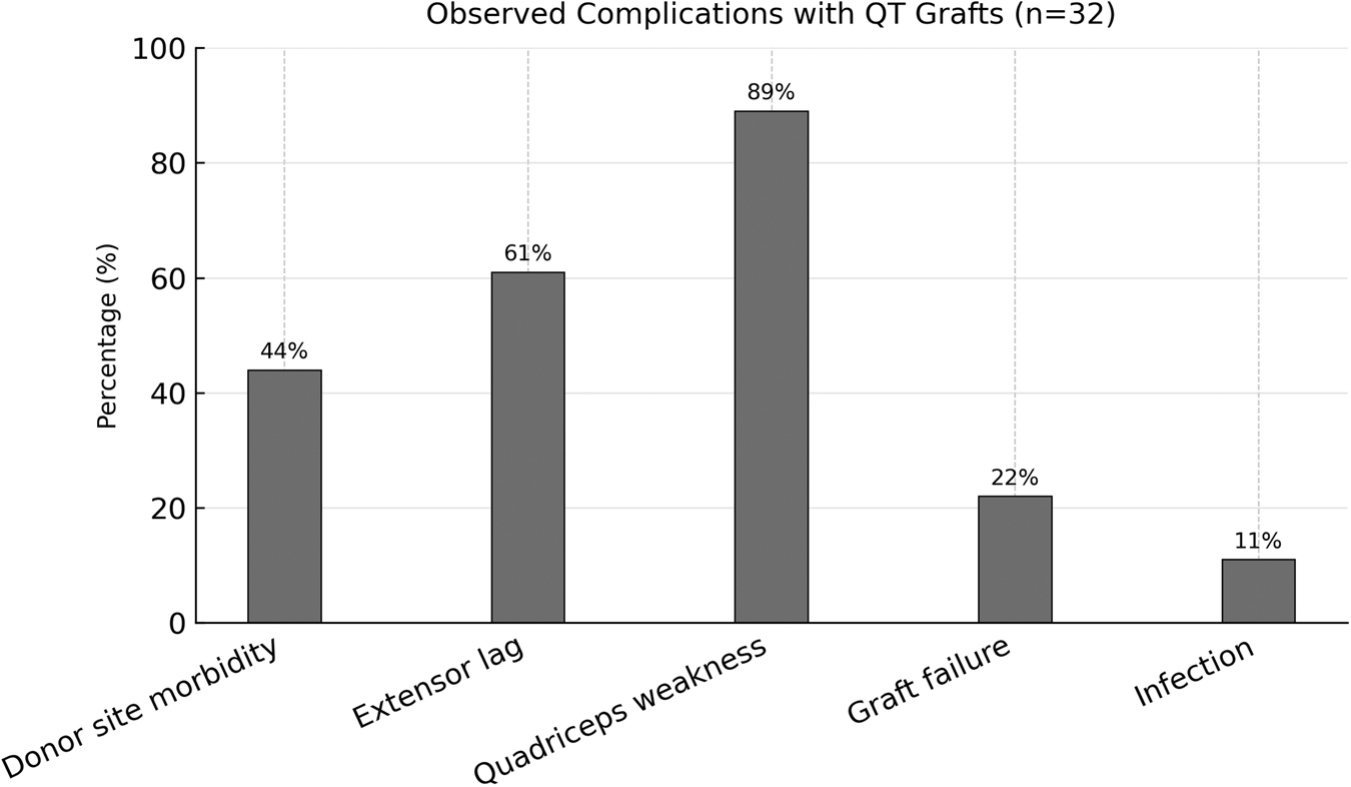
Most commonly observed complications following QT‐ACLR. ACLR, anterior cruciate ligament reconstruction; QT, quadriceps tendon.

Quadriceps weakness (89%, *n* = 28) and extensor lag (61%, *n* = 20) were the most frequent short‐term complications. Donor‐site morbidity was reported by 44% (*n* = 14), infection by 11% (*n* = 4) and graft failure by 22% (*n* = 7), the latter representing a general adverse outcome of ACL reconstruction rather than a QT‐specific complication (Figure 6).

**Figure 7:**
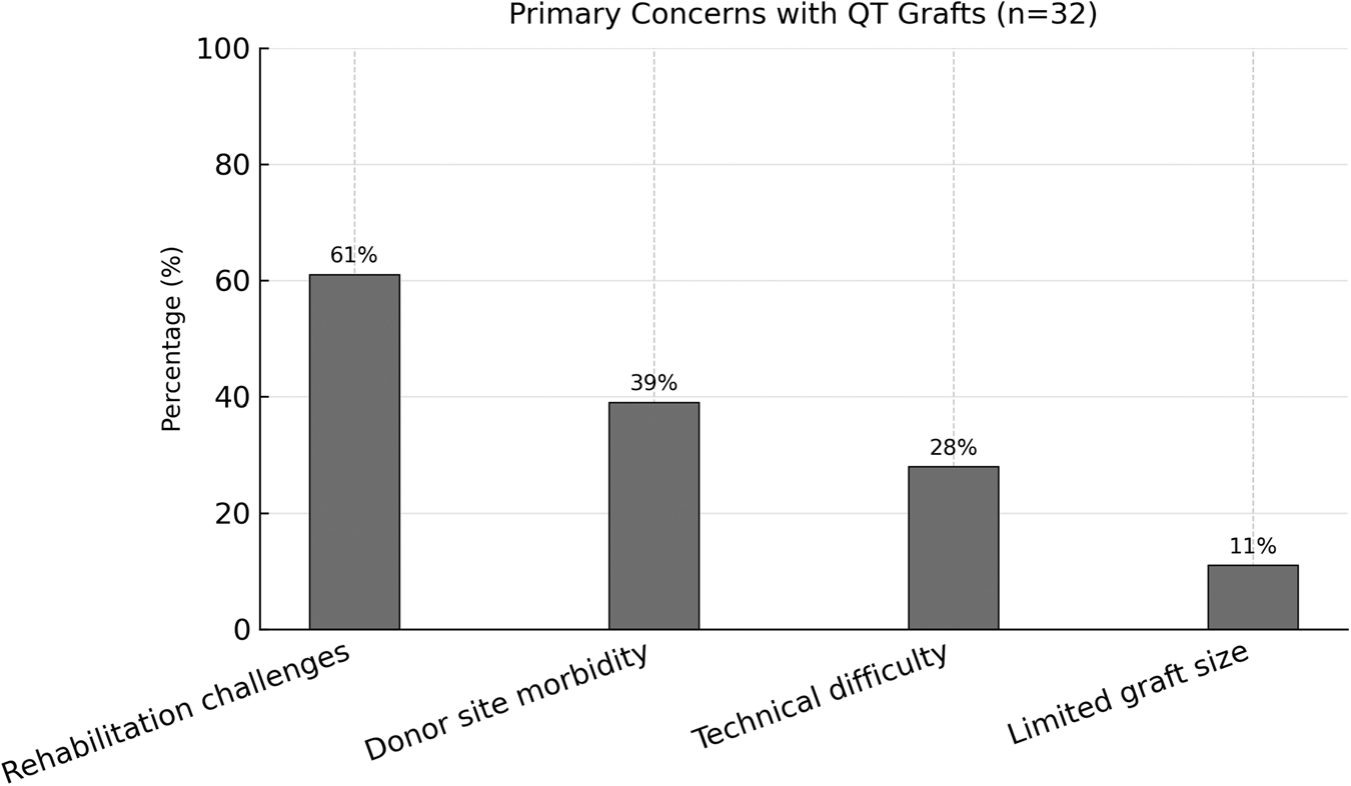
Primary concerns with the use of QT. QT, quadriceps tendon.

Regarding concerns with QT use, 61% (*n* = 20) mentioned rehabilitation challenges, 39% (*n* = 12) morbidity, 28% (*n* = 9) technical difficulty and 11% (*n* = 4) limited graft size. QT rupture after harvesting was reported by 11% (*n* = 4). Additionally, 33% (*n* = 11) had encountered that QT grafts were too short or narrow for ACLR (Figure 7).

## DISCUSSION

The most relevant finding of the present survey was the persistent heterogeneity in primary graft selection, even within an international cohort of surgeons with specific experience in QT‐ACLR. While 61% (*n* = 20) identified QT as their preferred graft, 39% (*n* = 12) favoured HT autograft, indicating that QT use, although common in this expert group, was not universal. This variability suggests that graft choice in contemporary ACLR remains influenced by individual surgical experience, patient factors and procedural context rather than uniform preference for a single autograft option. However, while BPTB and HT autografts remain widely popular and most commonly used worldwide, the growing preference for QT reflects expanding confidence in its anatomical, biomechanical and clinical advantages [3, 6, 34, 35, 36, 49]. Furthermore, within this heterogeneous landscape, QT demonstrated particularly strong acceptance in more complex settings. The adaptability of the QT was further emphasized by its widespread use in revision ACLR (89%) and multiligamentous reconstructions (72%) among experts. These findings are consistent with prior studies highlighting the graft's favourable length, diameter and harvesting versatility, especially when bone block inclusion is feasible [44, 48, 51, 52, 57, 58, 60]. Anatomical studies have shown that the central QT provides consistent width and thickness, even in smaller patients [23, 24, 59], further supporting its reliability across diverse populations. Furthermore, histologic investigations have also reinforced the distinctive structural profile of the QT [37]. Compared with hamstring tendons, which display higher fibroblast and vascular density, and the patellar tendon, which demonstrates a lower fibril–interstitium ratio, the QT is characterized by densely organized Type I collagen, lower proportions of Type III and V collagen and fewer elastic fibres [14].

Despite its advantages, some respondents reported concerns regarding graft length and diameter. However, biomechanical data support the adequacy of QT grafts as small as 8 mm, which have demonstrated failure loads exceeding 2500 N—well above the threshold required for ACLR [30]. To minimize arthrofibrosis, some authors recommended maintaining graft diameters around 9.0 mm, with an upper limit of 9.5 mm [15]. Importantly, the favourable biomechanical profile of the QT allows the safe use of smaller diameters within this range [5, 30, 38].

Clinical outcomes further reinforce these observations. Systematic reviews have reported that QT autografts provide results at least equivalent to HT and BPTB, with the additional benefit of reduced donor‐site morbidity, particularly less harvest‐site pain and kneeling discomfort when compared to BPTB grafts [2, 40, 46, 48] or lower rupture grafts in young active populations compared to HT autograft [36, 40, 42, 45, 47]. Registry‐based studies also confirm similar revision rates between QT and HT autografts in athletic cohorts [10]. Arthrofibrosis has also been raised as a concern in the literature; however, comparative studies have not demonstrated significant differences in secondary arthrofibrosis rates between graft options [20]. Johnson et al. [20] analysed a total of 1726 primary ACLR, comparing QT (*n* = 571) and BPTB (*n* = 1,155) autografts and found no significant difference in the rate of secondary arthrofibrosis between graft options (6.5% vs. 5.2%, *p* = 0.275).

In revision ACLR, QT has been consistently associated with favourable stability, satisfactory patient‐reported outcome measures (PROMs) and acceptable return‐to‐sport rates, with failure rates around 8% [33, 35, 36]. These data support the broad applicability of QT across both primary and revision settings, particularly in young, high‐demand athletes [36].

Nevertheless, donor‐site morbidity and rehabilitation challenges following QT harvest remain a concern [26, 29]. In this survey, transient quadriceps weakness and extensor lag were the most frequently reported short‐term complications, a finding consistent with clinical studies that document weakness during early rehabilitation, typically resolving within 3–6 months [17, 18, 22]. Meta‐analyses suggest that QT autografts yield superior knee flexion strength compared with HT, while BPTB and QT provide comparable outcomes at 6 and 12 months [16]. Conversely, HT may allow for faster recovery of extension strength early postoperatively, although this difference disappears by 12 months. Johnston et al. [21] reported that extensor strength deficits after QT‐based ACLR may persist up to 24 months, while Greiner et al. [13] identified female sex, older age and greater graft width as independent predictors of reduced extension symmetry. In contrast to these functional recovery considerations, severe structural extensor mechanism complications appear to be rare. Trasolini et al. [56], in a meta‐analysis of 28 studies, reported pooled proportions of QT rupture of 0.52% and patellar fracture of 2.03% following QT harvest, demonstrating that major structural extensor mechanism complications are uncommon. Taken together, these findings suggest that QT graft use is generally safe from a structural standpoint, but postoperative extensor mechanism recovery should be anticipated and discussed, and graft selection should therefore be individualized based on patient factors, functional demands and rehabilitation considerations [6, 33, 34, 49, 52].

Fixation technique represents another source of variability. The suspensory fixation was most frequently used on the femoral side, while tibial fixation was predominantly achieved with interference screws. Biomechanical studies indicate that both methods provide sufficient strength when applied correctly [8, 19]. However, differences in tunnel orientation, graft tensioning and surgical experience may influence outcomes. Arakgi et al. [1] compared three soft‐tissue suspensory fixation methods with bone‐block controls and reported greater displacement under cyclic loading in all soft‐tissue techniques, without identifying a clearly superior approach. In this context, the survey finding that 28% of respondents cited technical difficulty and 33% reported encountering QT grafts perceived as too short or narrow suggests that graft handling and fixation strategy remain relevant practical considerations, since in all–soft tissue QT constructs, the absence of a bone block may in some cases limit the applicability of interference screw fixation and increase reliance on suspensory fixation [1, 3, 8, 30]. Interestingly, the most frequently reported harvest approach remained relatively ‘conventional’, with 67% (*n* = 21) preferring a vertical incision and 56% (*n* = 18) using free‐hand harvest, suggesting that broader dissemination of minimally invasive instrument‐assisted techniques may be variable even among dedicated QT users. These findings outline the importance of meticulous surgical technique and surgical experience rather than harvesting and fixation methods alone.

Preoperative imaging of the QT, despite its potential value in surgical planning, remains underutilized among experts, with only a minority of respondents who reported routine use of MRI or ultrasound to evaluate graft thickness preoperatively. This is notable given prior work demonstrating that QT thickness correlates with patient size and that imaging may help identify grafts at risk of insufficient dimensions [25, 31, 59]. Wider adoption of imaging‐based preoperative planning could further enhance the predictability of graft selection.

Another interesting finding of this survey is that none of the participants chose BPTB autograft as primary autograft choice. Conversely, in a recent survey study including 68 high‐volume experienced surgeons of the Herodicus society, 58.6% of the colleagues preferred using BPTB option in their primary ACLR, whereas QT was the second most frequently used graft option [10]. This contrast shows the heterogeneity of graft preferences among experienced surgeons, but given the favourable outcomes consistently reported for the most widely used autograft options—the BPTB, HT and QT autografts, graft selection in clinical practice often depends more on individual surgeon preference, training and institutional culture rather than clear superiority of one option [11, 33, 40, 61]. Because the present cohort consisted of surgeons with a particular focus on QT techniques, graft‐preference survey data should be interpreted in the context of an expected selection effect. Notably, the fact that QT was not the primary graft choice for all respondents indicates that QT is regarded as an important option rather than a universally preferred solution across all patients, surgeons or clinical scenarios. The availability of multiple effective autograft options may therefore be considered advantageous, as it allows reconstruction strategies to be tailored to patient characteristics and surgeon expertise.

### Limitations

Several limitations of this study should be acknowledged. First, although this survey reflects the global perspectives of experienced high‐volume knee orthopaedic surgeons, the results represent expert opinion and not a formal consensus statement. The limited number of participants can be seen as a limitation as well. While the response rate was high, with 32 participants completing the survey, the sample was predominantly male (28 male and 4 female) and largely based in Europe and North America. Notably, there was no representation from Australia, and limited representation from Asia, Africa and South America, which may restrict the generalizability of the findings across all geographic regions. Some questions did not allow for further specification; that is, it remains unclear whether there are different preferences between surgeons primarily using all–soft tissue QT grafts versus QT grafts harvested with a bone block. Further, since the survey targeted an initiative focused on QT techniques, responses likely reflect a population with greater familiarity and interest in QT use than the broader ACL community. Accordingly, graft‐preference proportions should not be interpreted as population‐level usage rates, but rather as practice patterns and perceptions within this international QT‐focused cohort.

## CONCLUSION

This international survey shows that the QT autograft is a well‐established option in ACLR among experienced knee surgeons, particularly in revision and complex settings. At the same time, persistent variability in primary graft selection and reported rehabilitation and technical considerations indicate that QT use remains individualized within a spectrum of effective autograft choices.

## AUTHOR CONTRIBUTIONS

All authors have made substantial contributions to the conception and design, acquisition of data or analysis and interpretation of data.

## CONFLICT OF INTEREST STATEMENT

**Danko Dan Milinkovic**: Member of the Editorial Board of *Knee Surgery, Sports Traumatology, Arthroscopy* (*KSSTA*). **Mirco Herbort**: Reports relationships with Medacta International, Arthrex, Stryker and Enovis that include consulting or advisory roles and speaking/lecture fees; has received royalties from Medacta International. **Christian Fink**: Reports relationships with Medacta International and Karl Storz Endoskope that include consulting or advisory roles, speaking/lecture fees and royalties; reports a relationship with Zimmer Biomet that includes research funding grants; holds a patent with royalties paid to Karl Storz. **Volker Musahl**: ACL Study Group: Board or committee member. American Orthopaedic Society for Sports Medicine: Board or committee member. Arthrex, Inc.: Other financial or material support. International Society of Arthroscopy, Knee Surgery, and Orthopaedic Sports Medicine: Board or committee member. JISAKOS: Board or committee member. *Knee Surgery, Sports Traumatology, Arthroscopy*: Editorial or governing board. Newclip: Paid consultant. Ostesys: Stock or stock options. Smith & Nephew: Other financial or material support; Paid consultant. Springer: Publishing royalties, financial or material support. The remaining authors declare no conflict of interest.

## ETHICS STATEMENT

The authors have nothing to report.

## Supporting information

Revised_Appendix‐ Complete Poll Questions with Results.

## Data Availability

Raw data can be made available upon request.
