# Supplementary material for: Quadriceps tendon autograft for ACL reconstruction: A global survey from the International Quadriceps Tendon Interest Group
Source: J Exp Orthop. 2026 Mar 25;13(1):e70691. doi: 10.1002/jeo2.70691 (PMC13238833; doi:10.1002/jeo2.70691)
Supplement: Supplementary file 1 — Revised_Appendix‐ Complete Poll Questions with Results. [file JEO2-13-e70691-s001.docx]

**Appendix I - List of Questions divided into 5 Categories and Poll Results**

1. ***Demographics***
2. **How old are you?**

- <35: 6% (n=2)
- 35–45: 22% (n=7)
- 45–55: 61% (n=20)
- >55: 11% (n=4)

1. **Country of origin?**

- Europe: 56.3% (n=18)
- North America: 28.1% n=9
- South America: 6.3% (n=2),
- Asia: 6.3% (n=2)
- Africa 3.1% (n=1)
- Australia: 0

1. **Country where you practice?**
2. Europe: 56.3% (n=18)
3. North America: 28.1% n=9
4. South America: 6.3% (n=2),
5. Asia: 6.3% (n=2)
6. Africa 3.1% (n=1)
7. Australia: 0
8. **How many ACL reconstructions do you perform per year?**

- <50: 17% (n=5)
- 50–100: 22% (n=7)
- 101–150: 28% (n=9)
- >150: 33%22% (n=11)

1. **What is your primary graft choice for ACL reconstruction?**

- Hamstring tendons (HT): 39% (n=12)
- Bone-Patellar tendon-Bone (BTP): 0% (n=0)
- Quadriceps tendon (QT): 61% (n=20)
- Peroneus longus tendon: 0% (n=0)
- Allograft: 0% (n=0)
- Other: 0% (n=0)

1. **What surgical technique do you use for ACL reconstruction?**

- Transtibial: 0% (n=0)
- Transportal: 94% (n=30)
- Outside-In: 6% (n=2)

1. ***Indications and Diagnostic Considerations***
2. **For which procedures do you prefer using the QT autograft?** *Note: multiple answers* *allowed*

- Primary ACL reconstruction: 78% (n=25)
- Revision ACL reconstruction: 89% (n=28)
- Multiligamentous injuries: 72% (n=23)
- Other: 0% (n=0)

1. **In what percentage of your primary ACL reconstructions do you use the QT autograft?**

- 0–25%: 17% (n=5)
- 26–50%: 22% (n=7)
- 51–75%: 28% (n=9)
- 76–100%: 33% (n=11)

1. **Is there a specific patient population you prefer treating with QT-ACL reconstructions? ****Note: multiple answers* *allowed*

- All patients: 39% (n=12)
- Active athletes: 39% (n=12)
- Female football players: 39% (n=12)
- Alpine skiers: 17% (n=5)
- Other: 11% (n=4)

1. **Do you use QT grafts in skeletally immature patients?**

- Always: 28% (n=9)
- Often: 17% (n=5)
- Sometimes: 50% (n=16)
- Never: 6% (n=2)

1. **What are in your opinion the main advantages of the QT autograft? ****Note: multiple answers* *allowed*

- Morphological characteristics: 78% (n=25)
- Biomechanical properties: 100% (n=32)
- Availability: 61% (n=20)
- Other: 6% (n=2)

1. **What demographic factors play an important role in your consideration for using QT autograft? ****Note: multiple answers* *allowed*

- Age: 78% (n=25)
- Sex: 44% (n=14)
- Height: 17% (n=5)
- BMI: 22% (n=7)
- Profession: 78% (n=25)
- Level of activity: 89% (n=28)
- Nicotine abuse: 6% (n=2)
- Other: 6% (n=2)

1. **What clinical/radiographic factors play an important role in your consideration for QT use? ****Note: multiple answers* *allowed*

- Leg axis (valgus/varus): 33% (n=11)
- Tibial slope: 39% (n=12)
- Dynamic/functional valgus: 39% (n=12)
- Extensor apparatus strength: 44% (n=14)
- Other: 17% (n=5)

1. **What are the main (absolute or relative) contraindications for QT autograft? ****Note: multiple answers* *allowed*

- Previous quadriceps tendon injuries or repairs: 94% (n=30)
- Severe quadriceps atrophy or neuromuscular dysfunction: 89% (n=28)
- Extensive scarring or poor tissue quality: 67% (n=21)
- Metabolic diseases: 22% (n=7)
- Genetic diseases: 11% (n=4)
- Other: 6% (n=2)

1. **Do you measure QT thickness preoperatively using MRI or ultrasound?**

- Always: 28% (n=9)
- Often: 6% (n=2)
- Sometimes: 39% (n=12)
- Never: 28% (n=9)

1. **If you assess QT thickness, which measurement do you consider most critical? ****Note: multiple answers* *allowed*

- Thickness 3 cm proximal to patella: 39% (n=12)
- Cross-sectional area: 44% (n=14)
- Tendon length: 28% (n=9)
- Other: 22% (n=7)

1. ***Surgical Technique***
2. **What is your preferred skin incision orientation for QT harvesting?**

- Vertical: 67% (n=21)
- Horizontal: 33% (n=11)
- Oblique: 0% (n=0)
- No preference: 0% (n=0)

1. **Do you use harvesting instruments or free-hand scalpel technique?**

- Harvesting instruments: 44% (n=14)
- Free hand: 56% (n=18)

1. **Do you harvest the periosteal flap?**

- Always: 28% (n=9)
- Often: 28% (n=9)
- Sometimes: 22% (n=7)
- Never: 22% (n=7)

1. **Do you suture the tendon defect after harvesting?**

- Always: 94% (n=30)
- Often: 0% (n=0)
- Sometimes: 0% (n=0)
- Never: 6% (n=2)

1. **Do you harvest the QT with a bone block?**

- Always: 11% (n=4)
- Often: 22% (n=7)
- Sometimes: 33% (n=11)
- Never: 33% (n=11)

1. **Which femoral fixation methods do you primarily use for QT grafts? ****Note: multiple answers* *allowed*

- Interference screw: 17% (n=5)
- Adjustable button: 61% (n=20)
- Fixed button: 39% (n=12)
- Hybrid fixation: 0% (n=0)
- Other: 6% (n=2)

1. **Which tibial fixation methods do you primarily use for QT grafts? ****Note: multiple answers* *allowed*

- Interference screw: 72% (n=23)
- Adjustable button: 22% (n=7)
- Fixed button: 6% (n=2)
- Hybrid fixation: 28% (n=9)
- Other: 6% (n=2)

1. **In what percentage of your primary ACL-R do you perform an extra-articular lateral procedure (LEAP)?**

- 0–25%: 33% (n=11)
- 26–50%: 50% (n=16)
- 51–75%: 11% (n=4)
- 76–100%: 6% (n=1)

1. ***Rehabilitation and postoperative management***
2. **Do you modify your rehabilitation protocol when using QT grafts compared to other grafts?**

- Yes: 33% (n=11)
- No: 67% (n=21)

1. **If yes, what modifications do you implement? ****Note: multiple answers* *allowed*

- Altered weight-bearing: 0% (n=0)
- Modified ROM: 33% (n=11)
- Extended immobilization: 11% (n=4)
- Other: 67% (n=21)

1. ***Complications and Limitations***
2. **What complications have you observed with QT grafts? ****Note: multiple answers* *allowed*

- Donor site morbidity: 44% (n=14)
- Extensor lag: 61% (n=20)
- Quadriceps weakness: 89% (n=28)
- Graft failure: 22% (n=7)
- Infection: 11% (n=4)
- Other: 0% (n=0)

1. **What are your primary concerns with QT grafts? ****Note: multiple answers* *allowed*

- Donor site morbidity: 39% (n=12)
- Technical difficulty: 28% (n=9)
- Limited graft size: 11% (n=4)
- Rehabilitation challenges: 61% (n=20)

1. **Have you experienced QT rupture after harvest?**

- Yes: 11% (n=4)
- Never: 89% (n=28)

1. **Have you encountered a QT graft that was too short or narrow for ACL use?**

- Yes: 33% (n=11)
- Never: 67% (n=21)
